# Effects of sanitary pad distribution and reproductive health education on upper primary school attendance and reproductive health knowledge and attitudes in Kenya: a cluster randomized controlled trial

**DOI:** 10.1186/s12978-021-01223-7

**Published:** 2021-08-31

**Authors:** Karen Austrian, Beth Kangwana, Eunice Muthengi, Erica Soler-Hampejsek

**Affiliations:** 1 Poverty, Gender and Youth Program, Population Council-Kenya, PO Box 17643-00500, Nairobi, Kenya; 2 Barcelona, Spain

**Keywords:** Adolescent girls, Randomized controlled trial, Kenya, Menstrual health, Sexual and reproductive health

## Abstract

**Background:**

Adolescent girls’ risk of school dropout and reproductive health (RH) challenges may be exacerbated by girls’ attitudes toward their bodies and inability to manage their menstruation. We assessed effects of sanitary pad distribution and RH education on girls in primary grade 7 in Kilifi, Kenya.

**Methods:**

A cluster randomized controlled trial design was used. Eligible clusters were all non-boarding schools in three sub-counties in Kilifi County that had a minimum of 25 girls enrolled in primary grade 7. 140 primary schools, 35 per arm, were randomly assigned to one of four study arms: (1) control; (2) sanitary pad distribution; (3) RH education; or (4) both sanitary pad distribution and RH education. Outcomes were school attendance, school engagement, RH knowledge and attitudes, gender norms, and self-efficacy. For outcomes measured both at baseline and endline, difference-in-differences (DID) models were estimated and for outcomes without baseline data available, analysis of covariance models were used.

**Results:**

The study enrolled 3489 randomly selected girls in primary grade 7, with a mean age of 14.4 (SD 1.5). Girls in arms 2 and 4 received on average 17.6 out of 20 packets of sanitary pads and girls in arms 3 and 4 participated on average in 21 out of 25 RH sessions. Ninety-four percent of the baseline sample was interviewed at the end of the intervention with no differential attrition by arm. There was no evidence of an effect on primary school attendance on arm 2 (coefficient [coef] 0.37, 95% CI − 0.73, 1.46), arm 3 (coef 0.14, 95% CI − 0.99, 1.26) or arm 4 (coef 0.58, 95% CI − .37, 1.52). There was increased positive RH attitudes for girls in arm 3 (DID coef. 0.63, 95% CI 0.40–0.86) and arm 4 (DID coef. 0.85, 95% CI 0.64, − 1.07). There was also an increase in RH knowledge, gender norms and self-efficacy in arms 3 and 4.

**Conclusions:**

The findings suggest that neither sanitary pad distribution nor RH education, on their own or together, were sufficient to improve primary school attendance. However, as the RH education intervention improved RH outcomes, the evidence suggests that sanitary pad distribution and RH education can be positioned in broader RH programming for girls.

*Trial registration:* ISRCTN, ISRCTN10894523. Registered 22 August 2017—Retrospectively registered, http://www.isrctn.com/ISRCTN10894523

**Supplementary Information:**

The online version contains supplementary material available at 10.1186/s12978-021-01223-7.

## Plain language summary

Adolescent girls face a range of challenges that may compromise their chances of completing school or their sexual and reproductive health. These challenges can be even further complicated by girls’ feelings of shame about their bodies, in particular about menstruation, or their lack of sanitary products to help them manage menstruation. This study sought out to assess if providing girls in grade 7 in a rural, coastal area of Kenya with sanitary pads and sex education would alleviate some of those challenges. One hundred and forty schools were included in the study and 35 each were randomly assigned to one of the following program packages: (1) standard government provision of pads and health education; (2) regular monthly provision of sanitary pads; (3) sex education; or (4) both regular monthly provision of sanitary pads and sex education. The study found that none of the three program packages had an impact on school attendance, however those that participated in the sex education felt more positively about menstruation, knew more about sexual and reproductive health, had more equitable gender norms and were more self-confident at the end of the program. The study results show that addressing girls’ menstrual health challenges are important, but are better positioned as part of comprehensive sexuality education programs addressing stigma and shame associated with menstruation, access to menstrual products, inequitable gender norms and sexual and reproductive health knowledge gaps, as opposed to a girls education intervention.

**Supplementary Information:**

The online version contains supplementary material available at 10.1186/s12978-021-01223-7.

## Background

As girls enter puberty their experience of sexual and gender based violence, school dropout, and early marriage starts to increase [1]. According to several qualitative studies in Africa, these vulnerabilities are exacerbated by girls’ lack of knowledge of their bodies and rights, and their inability to safely and comfortably manage their menstruation [2–5].

Qualitative studies conducted in Kenya, and other countries in sub-Saharan Africa, have identified several challenges girls face in managing their menstruation, including lack of access to menstrual products and lack of accurate information about menstruation. The studies also clarified that neither teachers [6] nor mothers [2, 7] felt well placed to deliver information on menstruation, let alone a wider range of sexual and reproductive health topics. In additional qualitative studies, girls expressed that they missed school during their menses due to lack of menstrual products, fear of leaking blood on their uniforms and pain from menstrual cramps [3, 5, 8]. A sense of shame, discomfort and need for secrecy around the topic of menstruation was also a common theme [2, 4–6]. Finally, girls expressed that when they were menstruating they experienced anxiety and stress about staining their uniforms, giving off an odor or in general being found out to be menstruating that made it difficult for them to concentrate or participate fully in class [2, 5, 9]. The literature on the challenges linked to a lack of menstrual hygiene products and knowledge has been largely qualitative.

While several programs have previously been developed to address girls’ menstrual health management (MHM) needs in Kenya, as well as globally, few have been rigorously evaluated, and where evidence does exist on the effect of such programs on reproductive health (RH) and schooling outcomes, the results have been mixed. A 2013 systematic review of the literature on the effects of MHM programs concluded that while there was some evidence on the effect of MHM on psycho-social outcomes, the impact on RH outcomes was unclear. They also noted that quantitative evidence was lacking on the effects of MHM on reducing school absenteeism and that there was an absence of rigorous studies showing the impact of MHM on girls’ general health and well-being [10].

In 2016, Hennegan and Montgomery published an MHM-related systematic review that assessed the risk of bias in eight studies and synthesized the evidence on the effects of MHM interventions on educational and psychosocial outcomes for women and girls in low and middle income countries [11]. The authors outlined two dominant types of MHM intervention approaches: hardware, or the provision of physical objects useful for MHM, such as menstrual cups or sanitary pads; and software, or the provision of human and social capital through education and non-tangible benefits. The review found considerable risk of bias in these studies and overall weaknesses in study designs such as small sample sizes, inability to determine causation, non-random assignment to study arms and short follow-up periods. Therefore, the review concluded that while there are some indications of positive results, insufficient evidence existed for the effectiveness of MHM interventions.

Since that review, the evidence base on the link between menstruation, menstrual products and education and RH outcomes has increased. A cluster RCT in rural Western Kenya found that while provision of menstrual cups or sanitary pads was associated with reduced sexually transmitted infection (STI) risk, there was no association with school dropout [12]. An analysis of school attendance data from this study showed a positive impact on attendance due to sanitary pad distribution, however, that effect washed out in models that accounted for absence due to school transfer [13]. A quasi-randomized controlled trial implemented in Uganda found positive effects from distribution of reusable sanitary pads and puberty education, both alone and combined; however the results should be interpreted with caution as the study had poor participant retention and a lack of fidelity to the intervention [14]. A cross sectional study of girls aged 14–18 years in a rural area of Uganda showed associations between menstruation and school attendance [8]. Finally, an analysis of longitudinal data on adolescent health in India showed that, conditional on school enrollment, menstruation is not a significant predictor of school attendance [15], which is a similar finding to an earlier study in Malawi that did not find an effect of menstruation on school attendance [16].

Despite the increase in quantitative evidence on the impact of menstrual products on education outcomes, studies assessing the effect of combined hardware and software interventions are lacking. In addition, MHM interventions are often embedded within the education or water, sanitation and hygiene (WASH) fields, yet the case has more recently been made that MHM and puberty education should be seen as an entry into girls discussing their bodies, and from which more comprehensive conversations on RH could then take place [17]. Evidence on the combined effect of an MHM and RH intervention has the potential to move that case forward. Therefore, this study aims to assess the effects of a combined hardware and software intervention, integrating both MHM and broader RH content, on education and RH outcomes.

## Methods

### Setting

This study took place in Kilifi County, Kenya. Kilifi was selected for the study as it ranked low in both education and RH indicators: for example, the transition rate from primary to secondary was 40% in 2010 compared to the national rate of 72% [18]; further, around 22% of girls ages 15–19 have begun childbearing, as compared to the national average of 18% [19].

In Kenya, the school year starts in January and consists of three academic terms per year. Primary school is from grade 1 through grade 8, and secondary school is from grade 9 to grade 12. While universal primary education for girls has nearly been achieved, there remains significant variation at the county level, gaps in the transition to secondary school and challenges with pupil absenteeism [19, 20]. The Government of Kenya’s policy has committed to sanitary pad distribution in schools with the intention that girls receive an allocation of pads each term; however, in practice Government reports have acknowledged that the supply chain supporting this policy are weak [21] and evaluations have shown that distribution of sanitary pads to schools were not reliable, and girls were not assured of equitable pad provision [22].

### Data and study design

This study assessed the impact of the Nia Project via a longitudinal, cluster-randomized controlled trial in 140 public primary schools in three rural sub-counties (Magarini, Kaloleni and Ganze) of Kilifi County, Kenya.

Study schools were randomly assigned to one of the following four study arms:

1. Control group
2. Sanitary pads distribution (pads only)
3. Reproductive health education (RH only)
4. Sanitary pads distribution + reproductive health education (combined)

**Fig. 1:**
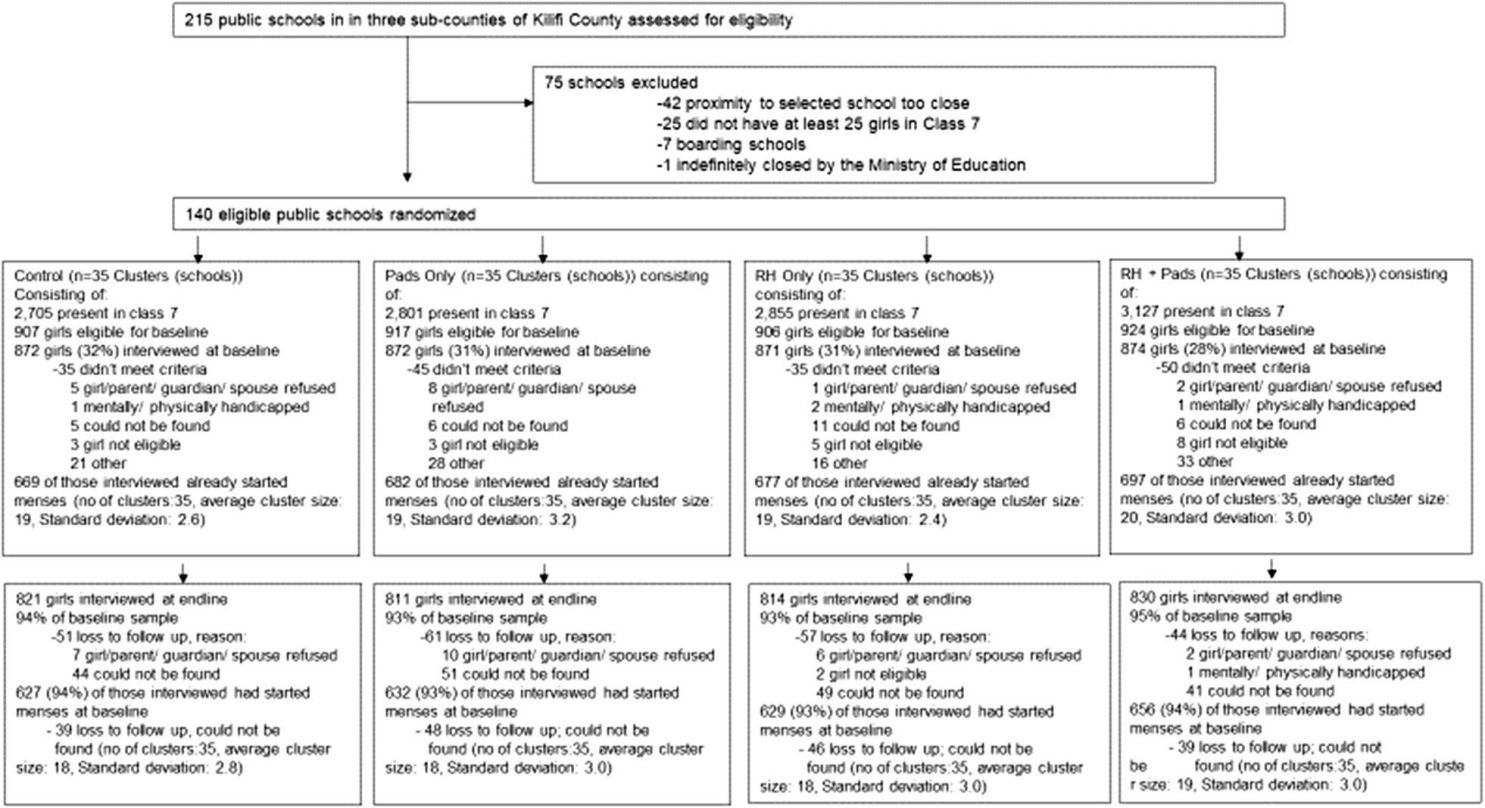
Sample flow

The sub-counties and schools were selected in collaboration with the Kilifi County Department of Education. Eligible clusters included all non-boarding schools in the three sub-counties with at least 25 girls enrolled in primary grade 7. A total of 215 schools were mapped, and a one kilometer buffer was created around each school. For schools with overlapping boundaries, one school was randomly selected resulting in a list of 173 schools. Enrollment and school type were verified for each school in the first quarter of 2017. Based on this exercise, 33 schools were excluded leaving a sample of 140 schools: 44 in Magarini, 50 in Kaloleni, and 46 in Ganze (see Fig. 1). All eligible schools (n = 140) were included in the study.

All girls enrolled in grade 7 in a randomized school were eligible for inclusion in the study sample. In schools with only 25 girls in grade 7, all girls were included in the research sample. In schools with a larger number of girls, 25 girls were randomly selected for the research sample and five additional girls were selected as alternates. A total of 3489 girls were interviewed as part of the baseline survey. All grade 7 girls, including those who were not in the research sample and those who had not yet started menstruating, were eligible to receive interventions in order to streamline program delivery. Grade 7 was selected as it would allow for observation of the transition to secondary school within the study timeframe.

The Nia Project included the following two components:

1. Sanitary pads: girls received, on a monthly basis for the entire duration of the project, one packet of ten disposable sanitary pads of ZanaAfrica’s Nia Teen brand. In addition, girls received two pairs of underwear at the start of the intervention, and an additional pair at the end of each subsequent school term.
2. RH education: a 25-session curriculum, *Nia Yetu*, was delivered by trained facilitators during girls-only health clubs held during time allocated for extra-curricular activities in schools. The curriculum covered a variety of topics including puberty, menstrual health management, reproductive systems, self-esteem, gender, human rights, power dynamics, sexual violence, assertiveness, decision making, relationships, teen pregnancy, STIs and HIV, peer pressure, drug use and conflict management. Girls also received a health magazine developed by ZanaAfrica, *Nia Teen*, designed to appeal to adolescent girls and convey core RH messaging through storytelling using aspirational personal stories, a relatable comic-style story, and activities. The magazine was distributed at the start of each school term for a five-term period. Each issue corresponded to the topics covered in the *Nia Yetu* curriculum that term.

Table 1 shows take-up of the two Nia Project components by study arms: girls in the pads only and combined arms received on average 17.5 out of 20 packets of sanitary pads and girls in the RH only and combined arms participated on average in 21 out of 25 RH sessions. Additional details on the Nia Project, theory of change, and study design have been published elsewhere [23].

**Table 1:** Nia project uptake among girls menstruating at baseline and interviewed at endline

	Arm 1 Control n = 627 (mean (SD))	Arm 2 Pads only n = 632 (mean (SD))	Arm 3 RH only n = 629 (mean (SD))	Arm 4 Pads and RH n = 656 (mean (SD))
Mean no. of pads received (target = 20)	0	17.5 (4.2)	0	17.5 (4.2)
Mean no. of underwear received (target = 6)		5.5 (1.3)		5.6 (1.3)
Mean no. of NIA magazines received (target = 5)	0	0.03 (0.2)	4.5 (1.2)	4.7 (1.1)
Mean no. of safe space sessions attended (target = 25)	0	0	20.7 (5.8)	21.2 (5.4)

Numbers shown in all columns to show potential for direct contamination in program implementation or through girls moving schools after program assignment

**Fig. 2:**
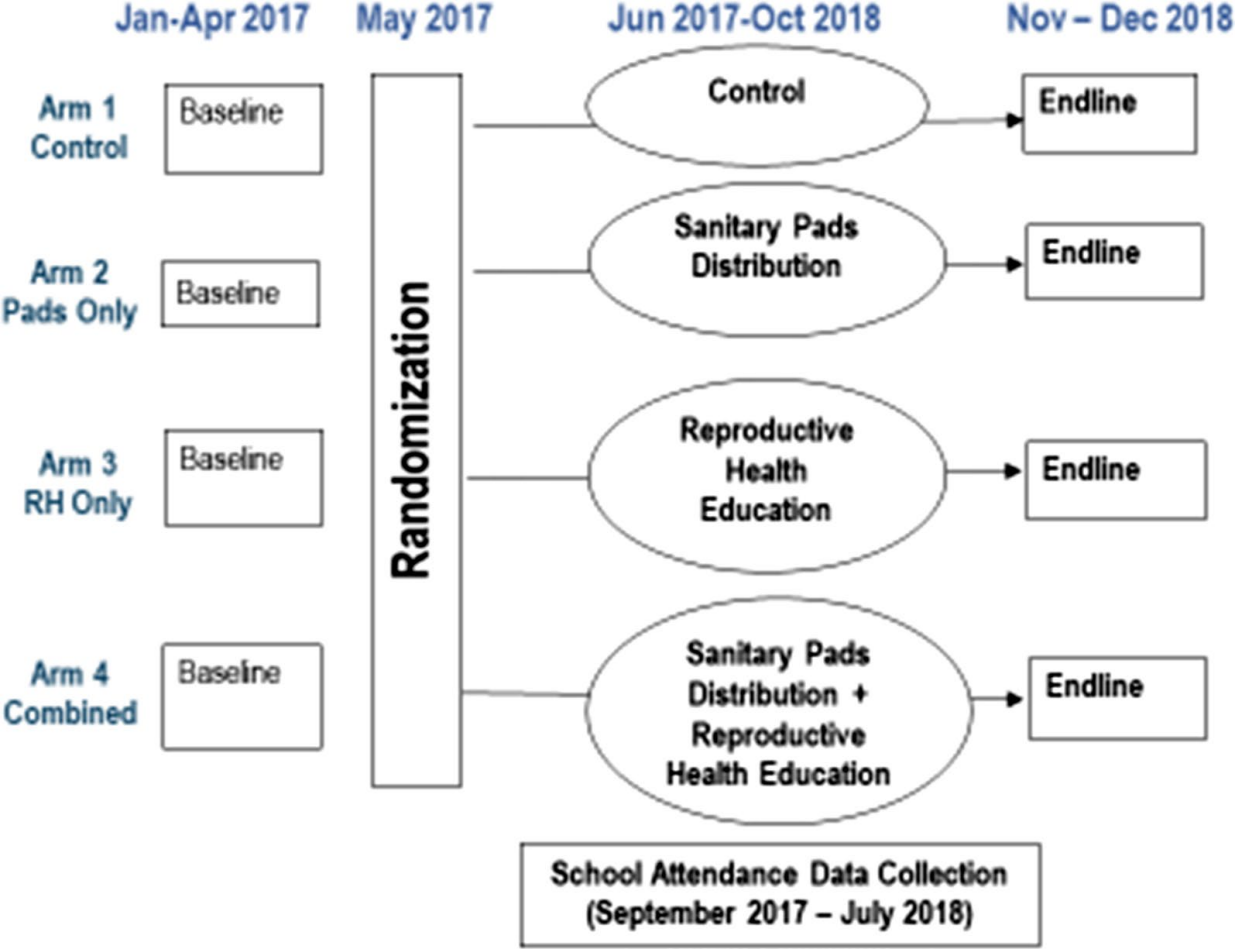
Study design

A baseline survey was conducted between January to April 2017, prior to the start of the intervention. Face-to-face interviews were carried out by a trained research assistant in Swahili and data was entered directly onto a tablet. Interviews were held in a private location to assure confidentiality, most commonly in the girls’ homestead or school (after school hours). School attendance tracking was carried out in two phases. First, an initial enrollment exercise took place in June 2017 where all students who were present in school were registered. Second, this registration list was updated at the start of each data collection term. Daily attendance was taken by community-based data collectors for a period of four weeks (20 consecutive school days) per term, starting in September 2017 through July 2018, for a total of 60 days of observation across three school terms. Girls who were registered during the enrollment period were entered as absent if they were absent on that particular day, or dropped out of school/transferred to another school during the observation period. Attendance data was entered as missing for girls who were not registered during the enrollment exercise. The intervention was completed in October 2018 and endline data was collected in November and December 2018 using the same technique as the baseline survey. All girls from the baseline sample were eligible for interview, regardless of schooling status. Figure 1 shows the sample flow by arm and Fig. 2 shows the study timeline.

### Randomization and masking

The unit of randomization was the school. At the completion of baseline data collection in each sub-county, prior to the start of the intervention, public lotteries were held and schools within that sub-county were randomly assigned to one of the four study arms. Both interviewers and respondents were blinded to study arm at baseline as it took place prior to randomization, but at endline the interviewers were aware of which schools had been assigned to each arm.

### Outcomes

Outcomes of interest related to education are: (i) school attendance, which was measured as the number of days a girl was attending school out of a total of 60 days. Mean school attendance was measured only for girls who remained in the same school from baseline to the end of the survey; and, (ii) school engagement measured with a 0–8 score constructed as the number of responses reflecting higher school engagement to eight agree/disagree survey items (e.g., “You are attentive in class”).

Menstruation management outcomes were binary measures including reporting having enough sanitary pads to comfortably manage menstruation and having leaked blood at school during menstruation. Outcomes of interest related to the RH education intervention include: (i) RH attitudes among girls who had started menstruating with a 0–12 score constructed as the number of responses reflecting a positive attitude to twelve agree/disagree survey items which captured girls’ feelings of shame, pride and comfort vis-à-vis menstruation (e.g., “I feel ashamed of my body when I have my period”); (ii) a pregnancy knowledge score with range 0–4 constructed as the number of correct answers to four pregnancy related items; (iii) whether a girl could spontaneously name a modern method of contraception; (iv) STI knowledge score with range 0–4 constructed as the number of correct answers to four STI related items; and (v) a HIV knowledge score with range 0–11 constructed as the number of correct answers to eleven HIV related items; (vi) gender norms in marriage with a 0–5 score constructed as the number of responses reflecting an equitable gender norm to five agree/disagree survey items (e.g., “If a husband and wife disagree on using family planning, the husband’s opinion should come first”); (vii) equitable adolescent gender norms with a 0–12 score constructed as the number of responses reflecting an equitable adolescent gender norm to twelve agree/disagree survey items (e.g., “Girls should be as independent as boys”); (viii) gendered sexual norms with a 0–5 score constructed as the number of responses reflecting an equitable sexual norm to five agree/disagree survey items (e.g., “Girls should cover up or they will attract unwanted sexual attention”); (ix) acceptability of intimate partner violence (IPV) was measured with an indicator on whether a girl finds IPV acceptable in any of five situations; and (x) general self-efficacy with a 0–10 score constructed as the number of responses reflecting self-efficacy to ten agree/disagree survey items (e.g., “You always manage to solve difficult problems if you try hard enough”). See Additional file 1: Table S1 for the list of survey items included in each outcome.

The following covariates were measured at baseline to assess balance across study arms: girls’ age, cognitive score with range 0–16 measured from a subset of Raven’s Coloured Progressive Matrices, math test score with a range 0–37 derived from a test including progressively harder problems, literacy score with a range 0–4 derived from reading sentences, using excerpts from the Uwezo Kenya National Learning Assessment [24], household wealth quintile, parental living status, and sub-county.

### Sample size and analytical sample

Based on findings of levels of detected differences in school attendance from previous studies conducted in Kenya and Ghana [25, 26], sample size calculations were conducted using Stata 15.1 to detect a minimum difference between study arms of 1.18 mean days of school missed over a 4-week period and a 10 percentage points increase in RH attitudes, assuming power of 0.80, significance level of 0.05, intra-cluster correlation (ICC) of 0.173 and a standard deviation (SD) of 3.57. A sample size of 35 clusters per arm and 20 girls per cluster at endline (25 girls per cluster at baseline, assuming a loss of 20% by endline) was needed. Therefore, 25 girls per school were included in the research sample. There was no oversampling to account for girls who had not started menstruating at baseline.

The analytical sample for this paper focuses on the sample of girls who had started menstruating at baseline and were re-interviewed at endline. Estimates including both menstruating non-menstruating girls at baseline are presented in Additional file 1: Tables S3–S6.

### Statistical analysis

To assess baseline balance across study arms among girls interviewed at endline, means and 95% confidence intervals (CIs) were estimated for the set of covariates described above as well as for outcome variables measured at baseline. An analysis was also conducted to assess bias due to potential differential attrition by study arms.

An intent-to-treat (ITT) approach was used to estimate the effect of each intervention arm relative to the control group. For outcomes measured both at baseline and endline, difference-in-differences (DID) models with girl-level fixed-effects were estimated to compare the change between baseline and endline for each intervention arm relative to the control group [27]. Formally, the following linear regression model was estimated for each outcome:

$$Y_{ijt} = \alpha_{0} + \alpha_{{1}} S^{2}_{ij} + \alpha_{{2}} S^{3}_{ij} + \alpha_{{3}} S^{4}_{ij} + \alpha_{{4}} t + \alpha_{{5}} S^{2}_{ij} t + \alpha_{{6}} S^{3}_{ij} t + \alpha_{{7}} S^{4}_{ij} t + {\text{a}}_{ij} + {\text{e}}_{ijt}$$

where *Y*_*ijt*_ is the outcome of interest for girl *i* in school *j* at time *t* (t = 0 is baseline), *S*^2^ is a dichotomous variable for a girl enrolled in a school assigned to Arm 2, *S*^3^ is a dichotomous variable for a girl enrolled in a school assigned to Arm 3, *S*^4^ is a dichotomous variable for a girl enrolled in a school assigned to Arm 4, a is a time-invariant individual effect and e is a random error. The coefficients related to the interactions between arms and time, α_5,_ α_6_ and α_7_, provide the DID estimates for each treatment arm relative to the control.

For outcomes with no comparable baseline data available, ANCOVA models were used to compare endline outcomes for each intervention arm relative to the control group while controlling for the following covariates measured at baseline: girls’ age, cognitive score, math and literacy scores, household wealth quintile, parental living status, and sub-county. Formally, the following linear regression model was estimated for each outcome:

$$Y_{ijt} = \beta_{0} + \beta_{{1}} S^{2}_{ij} + \beta_{{2}} S^{3}_{ij} + \beta_{{3}} S^{4}_{ij} + \beta_{{4}} X_{ij0} + {\text{e}}_{ijt}$$

where *X* is a vector of the control variables measured at baseline. The coefficients related to the treatment arms, β_1,_ β_2_ and β_3_, provide the estimates of the treatment effects for each treatment arm relative to the control.

All regressions were estimated with robust standard errors accounting for clustering at the school level. Statistical analysis was conducted using Stata 14.1.

## Results

Of the 3489 girls interviewed at baseline, 2725 (79%) had started menstruating. Of these, 2544 (93%) were successfully interviewed at endline. There was no differential attrition across arms. The majority of girls lost to follow up could not be physically located and therefore were not interviewed.

Girls in the analytical sample had a mean baseline age of 14.8 (SD:1.2). Girls’ skills were measured by their cognitive, math and literacy ability, and on average achieved scores of 55% (8.8/16 (SD:3.1)), 79% (29.4/37 (SD:4.0)) and 95% (mean 3.8/4 (SD:0.7)), respectively. At baseline, the majority of girls (82%) reported that both parents were alive (Table 2). Girls had moderately positive menstruation attitudes (mean score across arm: 7.6/12 (63.3%)). Girls were familiar with HIV (mean score across arms: 8.1/11 (74%)) and displayed lower levels of knowledge regarding when pregnancy is most likely to occur (mean score across arms: 1.8/4 (45%)), measures on norms and attitudes showed that equitable gender norms in marriage (mean score across arms: 3.2/5), equitable adolescent gender norms (mean score across arms 5.5/12), and gendered sexual norms (mean score across arms: 1.8/4). Girls general self-efficacy mean score across arms was 5.5/10, and the mean score across arms of how they perceived their engagement in school was 6.5 out of 8 (Table 4).

**Table 2:** Baseline characteristics among girls menstruating at baseline and interviewed at endline

	Arm 1 Control (n (%))	Arm 2 Pads Only (n (%))	Arm 3 RH Only (n (%))	Arm 4 Pads & RH (n (%))	Total n (%)
Sample of girls menstruating at baseline	669	682	677	697	2725
Sample of girls menstruating at baseline interviewed at endline N (%)	627 (93.7)	632 (92.7)	629 (92.9)	656 (94.1)	2544 (93.4)
Age (mean, SD)	14.8 (1.2)	14.7 (1.3)	14.8 (1.3)	14.8 (1.2)	14.8 (1.2)
Skills					
Cognitive (max score = 16) (mean (SD))	8.7 (3.1)	8.8 (3.1)	8.8 (3.2)	8.8 (3.1)	8.8 (3.1)
Math (max score = 37) (mean (SD))	29.4 (3.8)	29.2 (3.7)	29.3 (4.1)	29.7 (4.2)	29.4 (4.0)
Literacy (max score = 4) (mean (SD))	3.9 (0.5)	3.8 (0.7)	3.7 (0.8)	3.8 (0.6)	3.8 (0.7)
SES quintiles n (% in each quintile)					
Quintile 1 (most poor)	138 (22.0)	156 (24.7)	128 (20.4)	132 (20.1)	554 (21.8)
Quintile 2	141 (22.5)	131 (20.7)	135 (21.5)	137 (20.9)	544 (21.4)
Quintile 3	131 (20.9)	144 (22.8)	130 (20.7)	116 (17.7)	521 (20.5)
Quintile 4	106 (16.9)	107 (16.9)	112 (17.8)	139 (21.2)	464 (18.2)
Quintile 5 (least poor)	111 (17.7)	94 (14.9)	124 (19.7)	132 (20.1)	461 (18.1)
Parents living status n (%)					
Knows both parents alive	512 (81.7)	512 (81.0)	521 (82.8)	531 (81.0)	2076 (81.6)
Knows mother only alive	97 (15.5)	89 (14.1)	80 (12.7)	91 (13.9)	357 (14.0)
Knows father only alive	14 (2.2)	19 (3.0)	15 (2.4)	17 (2.6)	65 (2.6)
Knows no parent alive	4 (0.6)	12 (1.9)	13 (2.1)	17 (2.6)	46 (1.8)
Subcounty n (% in each subcounty)					
Ganze	204 (32.5)	212 (33.5)	220 (35.0)	211 (32.2)	847 (33.3)
Magarini	211 (33.7)	223 (35.3)	222 (35.3	232 (35.4)	888 (34.9)
Kaloleni	212 (33.8)	197 (31.2)	187 (29.7)	213 (32.5)	809 (31.8)

Table 3 shows results from the school attendance tracking instrument. Attendance was observed for the full 60 days for 2265 (89%) of girls in the analytical sample (no statistically significant difference by arm). On average, girls attended 55 days out of the 60 (SD: 7.6), and there was no difference between arms.

**Table 3:** School attendance outcomes from school attendance tracking instrument among girls menstruating at baseline and interviewed at endline

	Arm 1 Control	Arm 2 Pads only	Arm 3 RH only	Arm 4 Pads and RH
Respondents in analytical sample (N)	627	632	629	656
*Attendance was taken for all 60 days (% (N))*^*a*^	84.7 (531)	87.3 (552)	86.5 (544)	85.7 (562)
Intra-cluster correlation coefficient	0.000			
Coefficient (95% CI)	Reference	0.022 (− 0.047, 0.092)	0.015 (-0.049, 0.079)	0.007 (− 0.069, 0.083)
P-value		0.524	0.431	0.862
*Mean # of days attended (mean (SD))*^*a*^	55.6 (6.5)	56.0 (6.3)	55.8 (6.8)	56.2 (6.1)
Intra-cluster correlation coefficient	0.059			
Coefficient (95% CI)	Reference	0.37 (− 0.73, 1.46)	0.14 (− 0.99, 1.26)	0.58 (− 0.37, 1.52)
P-value		0.507	0.812	0.230
*Observed attendance*^*b*^* (% (N)):*	91.4 (619)	91.8 (625)	91.6 (622)	92.2 (647)
Intra-cluster correlation coefficient	0.056			
Coefficient (95% CI)	Reference	0.42 (− 1.94, 2.78)	0.28 (− 2.00, 2.57)	0.75 (− 0.97, 2.48)
P-value		0.725	0.806	0.389

The table reports post-intervention means for the control arm, and the estimated effect of the intent-to-treat for each study arm relative to the control arm. Difference-in-differences were estimated from regressions with girl-level fixed effects and robust standard errors accounting for clustering at the school level

Higher scores equate to higher knowledge and more positive/equitable norms and attitudes

^a^Attendance data only includes those who remained in the same school throughout the 60 days

^b^Differences at endline were estimated using ANCOVA. Regressions controlled for the following covariates measured at baseline: cognitive, math and literacy test scores, socio-economic quintile, age, parental living status, subcounty, and were estimated with robust standard errors accounting for clustering at the school level

Table 4 shows results from outcomes measured in the girl survey instrument. A positive increase was observed in girls reporting having enough pads in the pads only (DID coeff: 0.28 (95%CI: 0.20, 0.36)) and combined arms (DID coeff: 0.25 (95%CI:0.17, 0.33)), compared to the control. However, because girls in the control and RH only arms also had a significant increase in reporting having enough pads relative to baseline (36.5 and 43.3 percentage point increase for the control and RH only arms respectively, we carried out a post-hoc regression analysis to test if those increases explained the null attendance results. The post-hoc analysis showed no association between having enough pads and school attendance in girls who had started menstruating at baseline (coeff: 0.000354, 95% CI: − 01,000,585, 0.00129; p value = 0.457).

**Table 4:** Baseline and post intervention outcomes from survey among girls menstruating at baseline and interviewed at endline

	Arm 1 Control	Arm 2 Pads only	Arm 3 RH only	Arm 4 Pads and RH
*School engagement*				
School engagement^α^ (score 0–8):				
Baseline (N = 2544) (mean (SD) n)	6.5 (1.4) n = 627	6.6 (1.4) n = 632	6.5 (1.4) n = 629	6.5 (1.3) n = 656
Endline (N = 2432) (mean (SD) n)	6.8 (1.4) n = 593	6.8 (1.3) n = 606	6.8 (1.3) n = 600	6.9 (1.3) n = 633
Intra-cluster correlation coefficient	0.0214			
Coefficient (95% CI)	Reference	− 0.05 (− 0.33, 0.22)	0.14 (− 0.14, 0.42)	0.16 (− 0.14, 0.47)
P-value		0.703	0.329	0.294
*Menstruation management*				
Has enough pads (= 1):				
Baseline (N = 2544) (n (%) N)	136 (21.7), N = 627	126 (19.9), N = 632	119 (18.9), N = 629	155 (23.6), N = 656
Endline (N = 2541) (n (%) N)	350 (55.9), N = 626	521 (82.4), N = 632	371 (59.1), N = 628	543 (82.9), N = 655
Intra-cluster correlation coefficient	0.102			
Coefficient (95% CI)	Reference	0.28 (0.20, 0.36)	0.06 (− 0.03, 0.14)	0.25 (0.17, 0.33)
P-value		< 0.001	0.175	< 0.001
Reporting leaking (= 1):				
Baseline (N = 2544) (n (%) N)	213 (34.0), N = 627	240 (38.0), N = 632	235 (37.4), N = 629	263 (40.1), N = 656
Endline (N = 2432) (n (%) N)	161 (27.2), N = 593	125 (20.6), N = 606	143 (23.8), N = 600	138 (21.8), N = 633
Intra-cluster correlation coefficient	0.0139			
Coefficient (95% CI)	Reference	− 0.10 (− 0.18, − 0.03)	− 0.06 (− 0.13, 0.01)	− 0.11 (− 0.20, − 0.02)
P-value		0.005	0.118	0.014
*Reproductive health attitudes*				
Menstruation attitudes^#^ (score:0–12):				
Baseline (N = 2544) (mean (SD) n)	7.7 (1.7) n = 627	7.6 (1.8) n = 632	7.5 (1.8) n = 629	7.6 (1.7) n = 656
Endline (N = 2432) (mean (SD) n)	8.1 (1.6) n = 593	8.2 (1.6) n = 606	8.6 (1.6) n = 600	8.9 (1.6) n = 633
Intra-cluster correlation coefficient	0.0714			
Coefficient (95% CI)	Reference	0.16 (− 0.10, 0.41)	0.63 (0.40, 0.86)	0.85 (0.64, 1.07)
P-value		0.230	< 0.001	< 0.001
*Reproductive health knowledge*				
Pregnancy knowledge (score:0–4):				
Baseline (N = 2544) (mean (SD) n)	1.9 (0.9) n = 627	2.0 (0.8) n = 632	1.9 (0.9) n = 629	1.8 (0.9) n = 656
Endline (N = 2544) (mean (SD) n)	2.2 (0.9) n = 627	2.1 (0.9) n = 632	2.2 (0.9) n = 629	2.3 (0.9) n = 656
Intra-cluster correlation coefficient	0.0412			
Coefficient (95% CI)	Reference	− 0.15 (− 0.31, 0.01)	0.01 (− 0.16, 0.17)	0.18 (0.02, 0.34)
P-value		0.067	0.949	0.028
Can spontaneously name a method of modern contraception (= 1):				
Baseline (N = 2544) (n (%) N)	321 (51.2), n = 321	357 (56.5), n = 357	326 (51.8), n = 326	349 (53.2), n = 349
Endline (N = 2544) (n (%) N)	404 (64.4), n = 404	425 (67.2), n = 425	463 (73.6), n = 463	465 (70.9), n = 465
Intra-cluster correlation coefficient	0.0150			
Coefficient (95% CI)	Reference	− 0.03 (− 0.11, 0.06)	0.09 (0.01, 0.17)	0.04 (− 0.03, 0.12)
P-value		0.524	0.036	0.275
STI knowledge score (score:0–4):				
Baseline (N = 2544) (mean (SD) n)	0.4 (0.9) n = 627	0.5 (0.9) n = 632	0.4 (0.9) n = 629	0.4 (1.0) n = 656
Endline (N = 2544) (mean (SD) n)	1.2 (1.2) n = 627	1.2 (1.2) n = 632	1.5 (1.2) n = 629	1.5 (1.2) n = 656
Intra-cluster correlation coefficient	0.0536			
Coefficient (95% CI)	Reference	− 0.01 (− 0.19, 0.16)	0.31 (0.12, 0.49)	0.28 (0.10, 0.45)
P-value		0.873	0.002	0.002
HIV knowledge score (score:0–11):				
Baseline (N = 2544) (mean (SD) n)	7.8 (1.8) n = 627	8.0 (1.8) n = 632	8.0 (1.8) n = 629	8.0 (1.7) n = 656
Endline (N = 2544) (mean (SD) n)	8.4 (1.7) n = 627	8.3 (1.8) n = 632	8.5 (1.7) n = 629	8.4 (1.6) n = 656
Intra-cluster correlation coefficient	0.0215			
Coefficient (95% CI)	Reference	− 0.30 (− 0.61, 0.01)	− 0.05 (− 0.34. 0.24)	− 0.18 (− 0.43, 0 .08)
P-value		0.058	0.728	0.170
*Gender norms*				
Gender norms in marriage (score:0–5):				
Baseline (N = 2544) (mean (SD) n)	3.3 (1.1) n = 627	3.3 (1.2) n = 632	3.2 (1.2) n = 629	3.2 (1.1) n = 656
Endline (N = 2544) (mean (SD) n)	3.0 (1.2) n = 627	3.0 (1.2) n = 632	2.9 (1.2) n = 629	3.0 (1.2) n = 656
Intra-cluster correlation coefficient	0.0283			
Coefficient (95% CI)	Reference	0.01 (− 0.20, 0.22)	0.08 (− 0.13, 0.29)	0.10 (− 0.12, 0.31)
P-value		0.921	0.455	0.374
Equitable adolescent gender norms (score:0–12):				
Baseline (N = 2544) (mean (SD) n)	5.7 (1.9) n = 627	5.5 (2.0) n = 632	5.6 (1.9) n = 629	5.5 (2.0) n = 656
Endline (N = 2544) (mean (SD) n)	6.2 (1.8) n = 627	6.1 (1.8) n = 632	6.6 (1.9) n = 629	6.6 (1.8) n = 656
Intra-cluster correlation coefficient	0.0507			
Coefficient (95% CI)	Reference	0.08 (− 0.25, 0.40)	0.45 (0.15, 0.74)	0.57 (0.30, 0.85)
P-value		0.640	0.003	< 0.001
Gendered sexual norms (score:0–5):				
Baseline (N = 2544) (mean (SD) n)	1.8 (1.2) n = 627	1.8 (1.1) n = 632	1.7 (1.2) n = 629	1.8 (1.2) n = 656
Endline (N = 2544) (mean (SD) n)	1.9 (1.2) n = 627	1.9 (1.2) n = 632	2.2 (1.3) n = 629	2.2 (1.3) n = 656
Intra-cluster correlation coefficient	0.0640			
Coefficient (95% CI)	Reference	0.02 (− 0.18, 0.21)	0.46 (0.26, 0.65)	0.36 (0.15, 0.57)
P-value		0.878	< 0.001	0.001
Agrees with IPV (= 1):				
Baseline (N = 2544) (n (%) N)	458 (73.0), n = 458	458 (72.5), n = 458	456 (72.5), n = 456	465 (70.9), n = 465
Endline (N = 2544) (n (%) N)	454 (72.4), n = 454	461 (72.9), n = 461	477 (75.8), n = 477	470 (71.6), n = 470
Intra-cluster correlation coefficient	0.0344			
Coefficient (95% CI)	Reference	0.01 (− 0.06, 0.09)	0.04 (− 0.04, 0.12)	0.01 (− 0.05, 0.08)
P-value		0.772	0.323	0.687
*Self-efficacy*				
General self-efficacy (score:0–10):				
Baseline (N = 2544) (mean (SD) n)	5.1 (2.6) n = 627	5.2 (2.5) n = 632	5.2 (2.6) n = 629	5.5 (2.6) n = 656
Endline (N = 2544) (mean (SD) n)	5.7 (2.4) n = 627	5.6 (2.5) n = 632	6.6 (2.3) n = 629	6.3 (2.5) n = 656
Intra-cluster correlation coefficient	0.0444			
Coefficient (95% CI)	Reference	− 0.16 (− 0.54, 0.23)	0.80 (0.37, 1.24)	0.22 (− 0.20, 0.64)
P-value		0.420	< 0.001	0.301

The table reports post-intervention means for the control arm, and the estimated effect of the intent-to-treat for each study arm relative to the control arm. Difference-in-differences were estimated from regressions with girl-level fixed effects and robust standard errors accounting for clustering at the school level

Higher scores equate to higher knowledge and more positive/equitable norms and attitudes

^α^School engagement was only measured among respondents who were in school; at baseline all menstruating girls were in school, at endline 2432 girls were still in school

Girls also reported less leaking in both the pads only (DID coeff: − 0.10 (95%CI: − 0.18, − 0.03)) and combined arms (DID coeff: − 0.11 (95%CI: − 0.20, − 0.02)). A positive increase was observed in menstruation attitudes in both the RH only (DID coefficient (coeff): 0.63 (95%CI: 0.40, 0.86)) and combined arms (DID coeff: 0.85 (95%CI: 0.64, 1.07)). A comparison of estimates between intervention arms (combined v. pads only for menstruation outcomes; combined v. RH only for RH and norms outcomes) showed a larger effect size on RH attitudes in the combined arm as compared to the RH only arm (see Additional file 1: Table S2).

For the indicators measuring RH knowledge, an increase was observed in pregnancy knowledge in the combined arm (difference-in-difference (DID) coefficient: 0.18 (95% CI: 0.02, 0.34)). An increase was also observed in the percentage of girls who could spontaneously mention a modern method of contraception in the RH only arm (DID coefficient: 0.09 (95% CI: 0.01, 0.17)) and in STI knowledge in the RH only arm (DID coefficient: 0.31 (95%CI: 0.12, 0.49)) and combined arm (DID coefficient: 0.28 (95%CI: 0.10, 0.45)). For the indicators measuring norms and attitudes, positive increases were observed in gender norms measuring equitable adolescent gender norms in adolescents in both the RH only (DID coefficient:0.45 (95%CI:0.15, 0.74)) and combined arms (DID coefficient: 0.57 (95%CI: 0.30, 0.85)), as well as in norms on gendered sexual norms similarly in both the RH only (DID coefficient: 0.46 (95%CI:0.26, 0.65)) and combined arms (DID coefficient: 0.36 (95%CI:0.15, 0.57)). Finally, there was an increase observed in general self-efficacy in the RH only arm (DID coefficient: 0.80 (95%CI: 0.37, 1.24)).

A comparison between intervention arms showed a larger effect size on general self-efficacy in the RH only arm as compare to the combined arm (Additional file 1: Table S2). Supplementary tables show the impact of the intervention on the whole sample which includes menstruating and non-menstruating girls (Additional file 1: Tables S4–S6). There were no significant differences as compared to the restricted analytical sample.

## Discussion

In this cluster randomized trial of evaluating sanitary pad distribution and RH education in Kenya we see that neither intervention component, alone or in combination, improved school attendance among girls in primary grade 7. This finding is consistent with several recent quantitative studies rigorously examining the relationship between sanitary pad distribution and/or RH education on school attendance that also found no significant effect [13, 15].

There are a few hypotheses as to why the intervention did not translate into improved school attendance, mainly related to alternative reasons for why girls miss school. While it is likely, and supported in the qualitative literature, that girls experience physical and emotional discomfort during menstruation, it is possible that it is not a direct cause of absenteeism. Quantitative studies assessing reasons girls miss school mention poverty and lack of ability to pay school fees, low value placed on girls’ education and instability in households as the most common causes of absenteeism [28, 29], none of which are addressed via access to sanitary pads or RH education. Therefore, interventions addressing these causes might be better placed to have an effect on girls’ school attendance. In addition, this paper support the recent push to move away from a central focus on school attendance as the central outcome of MHM programs [30].

This trial did show that the RH education improved girls’ RH attitudes, in particular increasing the pride and comfort they feel vis-à-vis menstruation, as well as RH knowledge, endorsement of equitable gender norms and general self-efficacy. This is also consistent with a literature on comprehensive sexuality education and its ability, when implemented well and addressing gender and power, to improve RH outcomes [31]. Furthermore, regardless of whether sanitary pads or RH education translate into improved attendance at school, it is recognized that girls have the right to manage their menstruation safely and with dignity [32] and have the right to adequate sexual and reproductive health information [33].

There are a few limitations of the study that affect the external validity of the findings. First, the study was implemented in one rural setting—three sub-counties within one county. Therefore, while the findings could be relevant to other rural areas in the country and region, the findings cannot be generalized to urban areas. Second, the intervention was implemented with girls in primary grade 7 at the start of the study, which means that the findings cannot be generalized to girls earlier in primary school or in secondary school. Third, it is possible that the attendance taking activities heightened students’ and schools’ attention to attendance and inadvertently stimulated attendance. Fourth, the gender norms scales, although a significant effect was detected, had low alphas. Fifth, the study did not oversample for girls who had not yet started menstruating at baseline, results in a slightly smaller endline sample size than calculated for in the power estimates. Finally, the government pad distribution program in schools or other market factors such as a general reduction in the price of pads over time, although evenly distributed across arms, may have increased the access to pads in Kilifi, beyond a threshold that would show differences between arms. However, the post-hoc analysis conducted indicates that there was no association between access to pads and school attendance, independent of random assignment to study arm.

This study also has several strengths which allow it to make a significant contribution to the literature on the impact of MHM interventions on education and health outcomes. Key study design features—random assignment, a large sample size, 18-month follow up period and strong fidelity to the design during implementation—address key limitations of previous research, which include small sample sizes, inability to determine causation, non-random assignment to study arms, and shorter follow-up periods. This increases the relevance of the results as it contradicts previous, less rigorous studies assessing the same outcomes [8, 9, 14, 25].

## Conclusions

The results of this study suggest that in this specific context, neither sanitary pad distribution nor RH education, on their own or together, are sufficient to improve girls’ school attendance or engagement in class and therefore would caution again positioning MHM activities as girls education interventions. These activities would be better framed as part of comprehensive sexuality education programs aiming to address girls’ stigma and shame associated with menstruation, access to menstrual management products, inequitable gender norms and lack of knowledge key RH issues.

## Supplementary Information

**Additional file 1: Table S1.** Outcomes. **Table S2.** Differences in estimated effects of intention-to-treat across study arms among girls menstruating at baseline and interviewed at endline. **Table S3.** Nia project uptake among all girls interviewed at endline. **Table S4.** School attendance outcomes from school attendance tracking instrument among all girls interviewed at endline. **Table S5.** Baseline and post intervention outcomes from survey among all girls. **Table S6.** Differences in estimated effects of intention-to-treat across study arms among all girls.

## Data Availability

Study data in this paper, including de-identified individual data and data dictionary, will be made available open access upon publication. The data will be stored and available for downloading via the Adolescent Data Hub—http://popcouncil.org/girlcenter/adolescentdatahub/.

## Abbreviations

CI: Confidence interval
DID: Difference in differences
ICC: Intra-cluster correlation
IPV: Intimate partner violence
IRB: Institutional Review Board
ITT: Intent-to-treat
MHM: Menstrual Health Management
RCT: Randomized controlled trial
RH: Reproductive health
SD: Standard deviation
STI: Sexually transmitted infection
WASH: Water, sanitation and hygiene
