# Supplementary material for: Effects of sanitary pad distribution and reproductive health education on upper primary school attendance and reproductive health knowledge and attitudes in Kenya: a cluster randomized controlled trial
Source: Reprod Health. 2021 Aug 31;18:179. doi: 10.1186/s12978-021-01223-7 (PMC8406733; doi:10.1186/s12978-021-01223-7)
Supplement: Supplementary file 1 — Additional file 1: Table S1. Outcomes. Table S2. Differences in estimated effects of intention-to-treat across study arms among girls menstruating at baseline and interviewed at endline. Table S3. Nia project uptake among all girls interviewed at endline. Table S4. School attendance outcomes from school attendance tracking instrument among all girls interviewed at endline. Table S5. Baseline and post intervention outcomes from survey among all girls. Table S6. Differences in estimated effects of intention-to-treat across study arms among all girls. [file 12978_2021_1223_MOESM1_ESM.docx]

**Additional Tables**

**Table S1: Outcomes**

|  | Survey items | Survey instrument and  source |
| --- | --- | --- |
| Education outcomes |  |  |
| Observed school attendance | Observed daily by staff members for a period of four weeks per school term | School attendance tracking |
| School engagement  Score range: 0-8  Alpha: 0.50 | 1. You do not attend school regularly (1 point if ‘disagree’) 2. You are attentive in class (1 point if ‘agree’) 3. You rarely complete the tasks that are assigned to you in class (1 point if ‘disagree’) 4. It is difficult for you to complete your school work (1 point if ‘disagree’) 5. You feel comfortable participating in class (1 point if ‘agree’) 6. You do not feel confident answering questions in class (1 point if ‘disagree’) 7. It is difficult for you to pay attention in class (1 point if ‘disagree’) 8. You always complete your school work (1 point if ‘agree’) | Girl survey  Source: Malawi Schooling and Adolescent Study (2007) |
|  |  |  |
| Menstruation management  Has enough pads  Reported leaking  RH attitudes | Do you feel like you have enough pads to manage your period comfortably? (0 if ‘no’, 1 if ‘yes’)  Have you ever had any blood leak onto your body or clothes when you were at school? (0 if ‘no’, 1 if ‘yes’) | Girl survey  Girl survey |
| Menstruation attitudes  Score range: 0-12  Alpha: 0.40 | 1. I feel ashamed of my body when I have my period (1 point if ‘disagree’) 2. I'm uncomfortable in my body when I have my period (1 point if ‘disagree’) 3. It's important that I keep my period secret from anyone (1 point if ‘disagree’) 4. I feel proud that I have my periods (1 point if ‘agree’) 5. I wish I could never have periods (1 point if ‘disagree’) 6. A girl can engage in sports activities during her periods (1 point if ‘agree’) 7. It is easy to concentrate in class while on my periods (1 point if ‘agree’) 8. Periods are an illness (1 point if ‘disagree’) 9. A girl is unclean when she is on her period (1 point if ‘disagree’) 10. Menstruation is a normal process for girls (1 point if ‘agree’) 11. One should not bathe during menstruation (1 point if ‘disagree’) 12. A girl should not feel embarrassed if she stains her dress at school when on her periods (1 point if ‘agree’) | Girl survey  Source: ZanaAfrica |
| RH knowledge |  |  |
| Pregnancy knowledge  Score range: 0-4 | 1. Can pregnancy occur after kissing or hugging? 2. Is it possible for a woman get pregnant on the very first time she has sexual intercourse? 3. From one menstrual period to the next, are there certain days when a woman is more likely to become pregnant if she has sexual intercourse? 4. Is this time just before her period begins, during her period, right after her period has ended, or two weeks after her period? | Girls survey  Source:  DHS |
| Can spontaneously name a method of modern contraception | I would like to talk about family planning – the various ways or methods that a couple can use to delay or avoid a pregnancy. Which ways or methods have you heard about? Valid responses: pill, IUD, injectable, implant, male condom, female condom, emergency contraception | Girls survey  Source: DHS |
| STI knowledge  Score range: 0-4 | 1. Besides HIV, do you know of any other diseases that can be transmitted through sexual intercourse? 2. One can have STD/STIs without showing any symptoms 3. All STIs/STDs can only be transmitted via sexual intercourse 4. Condoms protect against most STDs/STIs | Girls survey  Source: Adolescent Girls Initiative – Kenya (2015) |
| HIV knowledge  Score range: 0-11 | 1. Now I would like to talk about something else. Have you ever heard of an illness called AIDS? 2. Can people reduce their chances of getting the AIDS virus by having just one uninfected sex partner who has no other sex partners? 3. Can people get the AIDS virus from mosquito bites? 4. Can people reduce their chance of getting the AIDS virus by using a condom every time they have sex? 5. Can people get the AIDS virus by sharing food with a person who has AIDS? 6. Can people reduce their chance of getting the AIDS virus by not having sexual intercourse at all? 7. Can people get the AIDS virus because of witchcraft or other supernatural means? 8. Is it possible for a healthy-looking person to have the AIDS virus? 9. Can the virus that causes AIDS be transmitted from a mother to her baby during pregnancy? 10. Can the virus that causes AIDS be transmitted from a mother to her baby during delivery? 11. Can the virus that causes AIDS be transmitted from a mother to her baby by breastfeeding? | Girls survey  Source: DHS |
| Gender norms |  |  |
| Gender norms in marriage  Score range: 0-5  Alpha: 0.37 | 1. Polygamous marriages are part of your culture, so they should continue (1 point if ‘disagree’) 2. It is better if a girls’ family arranges her marriage, than her choosing herself (1 point if ‘disagree’) 3. If a man doesn’t hit his wife, it means he doesn’t love her (1 point if ‘disagree’) 4. It is a man’s right to have sex with his wife whenever he wants (1 point if ‘disagree’) 5. If a husband and wife disagree on using family planning, the husband’s opinion should come first (1 point if ‘disagree’) | Girl survey  Source: Global Early Adolescent Study |
| Equitable adolescent gender norms  Score range: 0-12  Alpha: 0.65 | 1. Girls should be as independent as boys (1 point if ‘agree a lot’ or ‘agree a little’) 2. Girls should not go out with their friends unless an adult is present (1 point if ‘disagree a little’ or ‘disagree a lot’) 3. Girls feel they are more limited in what they can do and where they can go than boys (1 point if ‘disagree a little’ or ‘disagree a lot’) 4. Girls should have the same chances/opportunities as boys (1 point if ‘agree a lot’ or ‘agree a little’) 5. Girls should be able to move about as freely as boys (1 point if ‘agree a lot’ or ‘agree a little’) 6. It is as important for girls to do well in school as it is for boys (1 point if ‘agree a lot’ or ‘agree a little’) 7. Boys and girls should be equally responsible for household chores (1 point if ‘agree a lot’ or ‘agree a little’) 8. Girls should keep their thoughts to themselves and not say what they think in public (1 point if ‘disagree a little’ or ‘disagree a lot’) 9. Girls are expected to be humble (1 point if ‘disagree a little’ or ‘disagree a lot’) 10. Girls should not make too many demands on others (1 point if ‘disagree a little’ or ‘disagree a lot’) 11. A girl should say what she thinks even if it hurts someone’s feelings (1 point if ‘agree a lot’ or ‘agree a little’) 12. Girls who argue with their friends in public do not behave as ladies (1 point if ‘disagree a little’ or ‘disagree a lot’) | Girl survey  Source: Global Early Adolescent Study |
| Gendered sexual norms  Score range: 0-5  Alpha: 0.46 | 1. Girls should cover up or they will attract unwanted sexual attention (1 point if ‘disagree a little’ or ‘disagree a lot’) 2. Girls should be careful about the way they look so they are not seen as trying to seduce men (1 point if ‘disagree a little’ or ‘disagree a lot’) 3. It’s a girl’s fault if boys come onto them (1 point if ‘disagree a little’ or ‘disagree a lot’) 4. Girls wear short dresses to get boys’ attention (1 point if ‘disagree a little’ or ‘disagree a lot’) 5. Girls should be free to dress as they want (1 point if ‘agree a lot’ or ‘agree a little’) | Girl survey  Source: Global Early Adolescent Study |
| Agrees with IPV  Score range: 0-5  Alpha: 0.80 | Agrees a husband is justified in hitting or beating his wife in any of the following situations:   1. If she goes out without telling him? 2. If she neglects the children? 3. If she argues with him? 4. If she refuses to have sex with him? 5. If she burns the food? | Girl survey  Source: DHS |
| Self-efficacy |  |  |
| General self-efficacy score  Score range: 0-10  Alpha: 0.73 | 1. You always manage to solve difficult problems if you try hard enough (1 point if ‘agree’) 2. If someone is against you, you can still find ways to get what you want (1 point if ‘agree’) 3. It is easy for you to focus on your aims and accomplish your goals (1 point if ‘agree’) 4. You are confident that you could handle unexpected events very well (1 point if ‘agree’) 5. Because of the help you can get, you know how to manage unexpected situations (1 point if ‘agree’) 6. You can solve most problems if you make the necessary effort (1 point if ‘agree’) 7. You can remain calm when facing difficulties because you can rely on your own abilities (1 point if ‘agree’) 8. When you face a problem, you can usually find more than one solution (1 point if ‘agree’) 9. If you are in trouble, you can usually think of a solution (1 point if ‘agree’) 10. You can usually handle any situation that comes your way (1 point if ‘agree’) | Girl survey  Source: Generalized Self-Efficacy Scale |

**Table S2: Differences in estimated effects of intention-to-treat across study arms among girls menstruating at baseline and interviewed at endline**

|  | **(1)**  **Pads Only vs RH only**  **(p value)** | **(2)**  **Pads & RH vs Pads Only**  **(p value)** | **(3)**  **Pads & RH vs RH Only**  **(p value)** |
| --- | --- | --- | --- |
| **School outcomes** |  |  |  |
| Mean # of days attended school | -0.23 (0.695) | -0.21 (0.680) | -0.44 (0.388) |
| Observed attendance | 0.14 (0.919) | 0.334 (0.758) | 0.47 (0.657) |
| School engagement | -0.19 (0.192) | 0.22 (0.176) | 0.024 (0.887) |
| **Mensuration management** |  |  |  |
| Has enough pads | 0.22 (<0.001) | -0.033 (0.403) | 0.19 (<0.001) |
| Reporting leaking (%) | -0.047 (0.087) | -0.007 (0.856) | -0.053 (0.154) |
| **Reproductive health attitudes** |  |  |  |
| Menstruation attitudes | -0.49 (0.001) | 0.70 (<0.001) | 0.22 (0.054) |
| **Reproductive health knowledge** |  |  |  |
| Pregnancy knowledge | -0.15 (0.019) | 0.33 (<0.001) | 0.18 (0.009) |
| Can spontaneously name a method of modern contraception | -0.11 (0.005) | 0.069 (0.065) | -0.041(0.262) |
| STI knowledge score | -0.32 (0.001) | -0.029 (0.001) | -0.029 (0.761) |
| HIV knowledge score | -0.25 (0.124) | 0.12 (0.397) | -0.13 (0.346) |
| **Gender norms** |  |  |  |
| Gender norms in marriage | -0.070 (0.514) | 0.084 (0.428) | 0.014 (0.895) |
| Equitable adolescent gender norms | -0.37 (0.019) | 0.50 (0.001) | 0.13 (0.331) |
| Gendered sexual norms | -0.44 (<0.001) | 0.35 (0.001) | -0.096 (0.334) |
| Acceptability of IPV | -0.050 (0.756) | -0.043 (0.764) | -0.092 (0.527) |
| **Self-efficacy** |  |  |  |
| General self-efficacy | -0.96 (<0.001) | 0.38 (0.064) | -0.58 (0.011) |

Notes: The table reports differences across study arms from the estimates reported in Table 3. Column 1 compares the estimates for Pads only to RH, column 2 compares the estimates for Pads & RH to Pads only, and column 3 compares the estimates for Pads & RH to RH only. Minor differences in the reported differentials compared to the estimates presented in Table 3 are due to rounding.

**Table S3: Nia Project uptake Among All Girls Interviewed at Endline**

|  | **Arm 1**  **Control**  **n=821**  **(mean (SD))** | **Arm 2**  **Pads Only**  **n=811**  **(mean (SD))** | **Arm 3**  **RH Only**  **n=814**  **(mean (SD))** | **Arm 4**  **Pads & RH**  **n=830**  **(mean (SD))** |
| --- | --- | --- | --- | --- |
| Mean no. of pads received (target=20) | 0 (0) | 17.6 (4.0) | 0 (0) | 17.6 (4.1) |
| Mean no. of underwear received  (target=6) | 0 (0) | 5.6 (1.3) | 0 (0) | 5.6 (1.3) |
| Mean no. of NIA magazines received (target=5) | 0 (0) | 0.03 (0.2) | 4.6 (1.1) | 4.7 (1.0) |
| Mean no. of safe space sessions attended (target=25) | 0.001 (0.035) | 0.03 (0.17) | 21.1 (5.5) | 21.4 (5.3) |

Note: Numbers shown in all columns to show potential for direct contamination in program implementation or through girls moving schools after program assignment

**Table S4: School Attendance Outcomes from School Attendance Tracking Instrument among All Girls Interviewed at Endline**

|  | **Arm 1**  **Control** | **Arm 2**  **Pads Only** | **Arm 3**  **RH Only** | **Arm 4**  **Pads & RH** |
| --- | --- | --- | --- | --- |
| Respondents in research sample (N) | 821 | 811 | 814 | 830 |
| Attendance was taken for all 60 days (% (N))^£^ | 84.8 (696) | 88.2 (715) | 87.0 (708) | 87.2 (724) |
| Coefficient (95% CI) | Reference | 0.043 (-0.030, 0.12) | 0.030 (-0.038, 0.098) | 0.027 (-0.049, 0.10) |
| P-value |  | 0.242 | 0.389 | 0.487 |
| Mean # of days attended (mean (SD)) ^£^ | 55.9 (6.0) | 56.3 (5.9) | 55.1 (6.3) | 56.5 (5.8) |
| Coefficient (95 % CI) | Reference | 0.32 (-0.61, 1.24) | 0.16 (-0.78, 1.11) | 0.51 (-0.31,1.33) |
| P-value |  | 0.500 | 0.734 | 0.220 |
| Observed attendance ^+^ (% (N)) | 92.0 (811) | 92.4 (803) | 92.2 (806) | 92.6 (820) |
| Coefficient (95% CI) | Reference | 0.37 (-1.55, 2.28 ) | 0.20 (-1.75, 2.14) | 0.52 (-1.02, 2.06) |
| P-value |  | 0.707 | 0.842 | 0.505 |

Notes: The table reports post-intervention means for the control arm, and the estimated effect of the intent-to-treat for each study arm relative to the control arm. Difference-in-differences were estimated from regressions with girl-level fixed effects and robust standard errors accounting for clustering at the school level.

Higher scores equate to higher knowledge and more positive/equitable norms and attitudes.

^£^Attendance data only includes those who remained in the same school throughout the 60 days.

^+^Differences at endline were estimated using ANCOVA. Regressions controlled for the following covariates measured at baseline: cognitive, math and literacy test scores, socio-economic quintile, age, parental living status, subcounty, and were estimated with robust standard errors accounting for clustering at the school level.

Sample: 3,276 girls interviewed both at baseline and endline, unless otherwise noted.

^#^2,544 girls had started menstruating at baseline and 3,145 at endline.

**Table S5: Baseline and Post Intervention Outcomes from Survey among All Girls**

|  | **Arm 1**  **Control** | **Arm 2**  **Pads Only** | **Arm 3**  **RH Only** | **Arm 4**  **Pads & RH** |
| --- | --- | --- | --- | --- |
| **School Engagement^α^** (score 0-8): |  |  |  |  |
| Baseline (N=3,276) (mean(SD)n) | 6.6 (1.3), n=821 | 6.7 (1.4), n=811 | 6.5 (1.3), n=814 | 6.6 (1.3), n=830 |
| Endline (N=3,163) (mean(SD)n) | 6.9 (6.9), n=787 | 6.9 (1.3), n=785 | 6.9 (1.3),n=785 | 7.0 (1.2), n=806 |
| Coefficient (95% CI) | Reference | -0.052 (-0.32, 0.22) | 0.119 (-0.14, 0.37) | 0.121 (-0.16, 0.40) |
| P-value |  | 0.705 | 0.356 | 0.390 |
| **Menstruation Management** |  |  |  |  |
| Has enough pads (=1): |  |  |  |  |
| Baseline (N=2,544) (n(%)N) | 136 (21.7), N=627 | 126 (19.9), N=632 | 119 (18.9), N=629 | 155 (23.6), N=656 |
| Endline (N=3,141) (n(%)N) | 453 (58.2), N=778 | 645 (83.0), N=777 | 488 (62.2), N=784 | 675 (84.2), N=802 |
| Coefficient (95% CI) | Reference | 0.28 (0.20, 0.36) | 0.06 (-0.03, 0.14) | 0.25 (0.17, 0.33) |
| P-value |  | <0.001 | 0.175 | <0.001 |
| Reporting leaking (=1): |  |  |  |  |
| Baseline (N=2,544) (n(%)N) | 213 (34.0), N=627 | 240 (38.0), N=632 | 235 (37.4), N=629 | 263 (40.1), N=656 |
| Endline (N=3,032) (n(%)N) | 196 (26.3), N=746 | 142 (18.9), N=751 | 168 (22.2), N=756 | 159 (20.4), N=779 |
| Coefficient (95% CI) | Reference | (-0.18, -0.03) | (-0.13, 0.015) | (-0.20, -0.02) |
| P-value |  | 0.005 | 0.118 | 0.014 |
| If started menstruation, **Reproductive health attitudes**# (score:0-12): |  |  |  |  |
| Baseline (N=2,544) (mean(SD)n) | 7.7 (1.7), n=627 | 7.6 (1.8), n=632 | 7.5 (1.8), n=629 | 7.6 (1.8), n=656 |
| Endline (N=3,032) (mean(SD)n) | 8.2 (1.6), n=746 | 8.4 (1.6), n=751 | 8..7 (1.6), n= 756 | 8.9 (1.5), n=779 |
| Coefficient (95% CI) | Reference | 0.16 (-0.10, 0.41) | 0.63 (0.40, 0.86) | 0.85 (0.64, 1.06) |
| P-value |  | 0.230 | <0.001 | <0.001 |
| **Reproductive health knowledge** |  |  |  |  |
| Pregnancy knowledge (score:0-4): |  |  |  |  |
| Baseline (N=3,276) (mean(SD)n) | 1.8 (0.9), n=821 | 1.9 (0.9), n=811 | 1.8 (0.9), n=814 | 1.8 (0.9), n=830 |
| Endline (N=3,276) (mean(SD)n) | 2.2 (0.9), n=821 | 2.1 (.09), n=811 | 2.2 (0.9) , n=814 | 2.4 (1.0) , n=830 |
| Coefficient (95% CI) | Reference | -0.14 (-0.28, 0.003) | 0.08 (-0.07, 0.23) | 0.22 (0.08, 0.37) |
| P-value |  | 0.055 | 0.312 | 0.003 |
| Can spontaneously name a method of modern contraception (=1): |  |  |  |  |
| Baseline (N=3,276) (n(%)N) | 405 (49.3), N=821 | 438 (54.0), N=811 | 392 (48.2), N=814 | 419 (54.0), N=830 |
| Endline (N=3,276) (n(%)N) | 526 (64.1), N=821 | 521 (64.2), N=811 | 573 (70.4), N=814 | 572 (68.9), N=830 |
| Coefficient (95% CI) | Reference | -0.05 (-0.12, 0.03) | 0.08 (0.01, 0.14) | 0.04 (-0.03, 0.11) |
| P-value |  | 0.248 | 0.035 | 0.311 |
| STI knowledge score (score:0-4): |  |  |  |  |
| Baseline (N=3,276) (mean(SD)n) | 0.4 (0.9), n=821 | 0.5 (1.0), n=811 | 0.4 (1.0), n= 814 | 0.4 (1.0), n=830 |
| Endline (N=3,276) (mean(SD)n) | 1.3 (1.2), n=821 | 1.3 (1.2), n=811 | 1.5 (1.2), n=814 | 1.6 (1.2), n=830 |
| Coefficient (95% CI) | Reference | -0.08 (-0.24, 0.08) | 0.25 (0.07, 0.43) | 0.27 (0.10, 0.44) |
| P-value |  | 0.339 | 0.008 | 0.002 |
| HIV knowledge score (score:0-11): |  |  |  |  |
| Baseline (N=3,276) (mean(SD)n) | 7.9 (1.9), n=821 | 7.9 (1.8), n=811 | 7.9 (1.9), n=814 | 8.0 (), n=830 |
| Endline (N=3,276) (mean(SD)n) | 8.5 (1.6), n=821 | 8.3 (1.7), n=811 | 8.5 (1.7), n=814 | 8.5 (1.6), n=830 |
| Coefficient (95% CI) | Reference | -0.22 (-0.51, 0.07) | 0.01 (-0.27, 0.29) | -0.13 (-0.38, 0.12) |
| P-value |  | 0.132 | 0.965 | 0.303 |
| **Gender norms** |  |  |  |  |
| Gender norms in marriage (score:0-5): |  |  |  |  |
| Baseline (N=3,276) (mean(SD)n) | 3.4 (1.2), n=821 | 3.4 (1.2), n=811 | 3.2 (1.3), n=814 | 3.3 (1.1), n=830 |
| Endline (N=3,276) (mean(SD)n) | 3.1 (1.2), n=821 | 3.1 (1.2), n=811 | 3.0 (1.2), n=814 | 3.1 (1.2), n=830 |
| Coefficient (95% CI) | Reference | -0.022 (-0.022, 0.17) | 0.07 (-0.15, 0.29) | 0.11 (-0.094, 0.32) |
| P-value |  | 0.821 | 0.516 | 0.281 |
| Equitable adolescent gender norms (score:0-12): |  |  |  |  |
| Baseline (N=3,276) (mean(SD)n) | 5.7 (2.0), n=821 | 5.6 (2.0), n=811 | 5.7 (1.9), n=814 | 5.6 (2.0), n=830 |
| Endline (N=3,276) (mean(SD)n) | 6.35 (1.9), n=821 | 6.14 (1.9), n=811 | 6.72 (1.9), n=814 | 6.696 (1.9), n=830 |
| Coefficient (95% CI) | Reference | -0.05 (-0.35, 0.25) | 0.42 (0.14, 0.69) | 0.47 (0.20, 0.74) |
| P-value |  | 0.724 | 0.004 | 0.001 |
| Gendered sexual norms (score:0-5): |  |  |  |  |
| Baseline (N=3,276) (mean(SD)n) | 1.8 (1.2), n=821 | 1.8 (1.1), n=811 | 1.7 (1.2), n=814 | 1.7 (1.2), n=830 |
| Endline (N=3,276) (mean(SD)n) | 1.9 (1.2), n=821 | 1.9 (1.2), n=811 | 2.3 (1.3), n=814 | 2.2 (1.3), n=830 |
| Coefficient (95% CI) | Reference | -0.01 (-0.20, 0.18) | 0.41 (0.23, 0.59) | 0.37 (0.18, 0.56) |
| P-value |  | 0.942 | <0.001 | <0.001 |
| Agrees with IPV (=1): |  |  |  |  |
| Baseline (N=3,276) (n(%)N) | 598 (72.8), N=821 | 578 (71.3), N=811 | 588 (72.2), N=814 | 573 (69.0), N=830 |
| Endline (N=3,276) (n(%)N) | 586 (71.4), N=821 | 571 (70.4), N=811 | 602 (74.0), N=814 | 575 (69.3), N=830 |
| Coefficient (95% CI) | Reference | 0.01 (-0.05, 0.08) | 0.03 (-0.04, 0.11) | 0.02 (-0.05, 0.09) |
| P-value |  | 0.874 | 0.397 | 0.632 |
| **Self-efficacy** |  |  |  |  |
| General self-efficacy (score:0-10): |  |  |  |  |
| Baseline (N=3,276) (mean(SD)n) | 5.1 (2.7), n=821 | 5.3 (2.5), n=811 | 5.2 (2.6), n=814 | 5.4 (2.6), n=830 |
| Endline (N=3,276) (mean(SD)n) | 5.7 (2.4), n=821 | 5.7 (2.5), n=811 | 6.5 (2.3), n=814 | 6.4 (2.4), n=830 |
| Coefficient (95% CI) | Reference | -0.21 (-0.56, 0.15) | 0.85 (0.42, 1.28) | 0.35 (-0.06, 0.74) |
| P-value |  | 0.251 | <0.001 | 0.090 |

Notes: The table reports post-intervention means for the control arm, and the estimated effect of the intent-to-treat for each study arm relative to the control arm. Difference-in-differences were estimated from regressions with girl-level fixed effects and robust standard errors accounting for clustering at the school level.

Higher scores equate to higher knowledge and more positive/equitable norms and attitudes.

^+^Differences at endline were estimated using ANCOVA. Regressions controlled for the following covariates measured at baseline: cognitive, math and literacy test scores, socio-economic quintile, age, parental living status, subcounty, and were estimated with robust standard errors accounting for clustering at the school level.

Sample: 3,276 girls interviewed both at baseline and endline, unless otherwise noted.

^#^2,544 girls had started menstruating at baseline and 3,145 at endline.

^α^School engagement was only measured among respondents who were in school; at baseline all girls were in school, at endline 3,163 girls were still in school.

**Table S6: Differences in estimated effects of intention-to-treat across study arms among all girls**

|  | **(1)**  **Pads Only vs RH only**  **(p value)** | **(2)**  **Pads & RH vs Pads Only**  **(p value)** | **(3)**  **Pads & RH vs RH Only**  **(p value)** |
| --- | --- | --- | --- |
| **School outcomes** |  |  |  |
| Mean # of days attended school | -0.15 (0.746) | -0.19 (0.644) | -0.35 (0.403) |
| Observed attendance | -0.17 (0.875) | -0.16 (0.860) | -0.33 (0.720) |
| School engagement | -0.17 (0.274) | -0.003 (0.985) | 0.17 (0.246) |
| **Menstruation management** |  |  |  |
| Has enough pads | -0.22 (<0.001) | 0.033 (0.403) | -0.191 (<0.001) |
| Reporting leaking | 0.047 (0.087) | 0.007 (0.856) | 0.053 (0.154) |
| **Reproductive health attitudes** |  |  |  |
| Menstruation attitudes | 0.48 (0.001) | -0.70 (<0.001) | -0.22 (0.054) |
| **Reproductive health knowledge** |  |  |  |
| Pregnancy knowledge | 0.22 (0.001) | -0.36 (<0.001) | -0.15 (0.029) |
| Can spontaneously name a method of modern contraception | 0.12 (0.001) | -0.08 (0.024) | 0.04 (0.234) |
| STI knowledge score | 0.33 (<0.001) | -0.35 (<0.001) | -0.02 (0.836) |
| HIV knowledge score | 0.23 (0.136) | -0.09 (0.511) | 0.14 (0.305) |
| **Gender norms** |  |  |  |
| Gender norms in marriage | -0.13 (0.149) | -0.042 (0.698) | 0.095 (0.347) |
| Equitable adolescent gender norms | 0.49 (0.001) | -0.50 (<0.001) | -0.01 (0.937) |
| Gendered sexual norms | 0.41 (<0.001) | -0.38 (<0.001) | 0.03 (0.707) |
| Acceptability of IPV | 0.04 (0.765) | 0.03 (0.851) | 0.07 (0.607) |
| **Self-efficacy** |  |  |  |
| General self-efficacy | 1.05 (<0.001) | -0.55 (0.004) | 0.50 (0.027) |

Notes: The table reports differences across study arms from the estimates reported in Table A2. Column 1 compares the estimates for Pads only to RH, column 2 compares the estimates for Pads & RH to Pads only, and column 3 compares the estimates for Pads&RH to RH only. Minor differences in the reported differentials compared to the estimates presented in Table A2 are due to rounding.
